# Editorial: Novel insights into CAR T-cell associated neurotoxicity

**DOI:** 10.3389/fneur.2023.1322843

**Published:** 2023-11-07

**Authors:** Stella Bouziana

**Affiliations:** Department of Haematology, King's College Hospital NHS Foundation Trust, London, United Kingdom

**Keywords:** CAR T-cells, ICANS, neurotoxicity, hematological malignancies, solid tumors

Over the last few years, technological advances in genetic engineering have resulted in the emergence of the very promising chimeric antigen receptor (CAR) T-cell therapies. CAR T-cell therapies constitute a milestone in current cell and gene therapies and it seems that they will revolutionize the fields of Hematology and Oncology. Since the first introduction of CAR T-cell technology in the late 1980s, major steps in the refinement of CAR T-cell production have been achieved leading to the first US Food and Drug Administration (FDA) approved commercial product in 2017 for the management of highly aggressive B-cell hematological malignancies. Both pivotal clinical trials and real world data have demonstrated unprecedented response rates in relapsed/refractory hematological cancers, covering the unmet need of treatment of patients with no other therapeutic options and enriching the treatment armamentarium for these challenging to treat patients (1).

Currently, the FDA has approved six CAR T-cell commercial products for the treatment of highly aggressive relapsed/refractory hematological malignancies, with the treatment indications gradually expanding while bringing CAR T-cells to earlier lines of treatment. In the meantime, extensive research is underway investigating the safety and efficacy of CAR T-cell therapies in solid tumors, with thousands of clinical trials currently running across the globe (2). Additionally, CAR T-cell therapies have been investigated in the management of other disease settings apart from malignancies, including autoimmune and infectious diseases, with very encouraging results so far (3).

However, despite the great success in achieving remissions, CAR T-cell therapies are still accompanied by great challenges. CAR T-cells can cause major toxicities hampering their broad application (4). The most frequently encountered toxicities include cytokine release syndrome (CRS) and immune effector cell-associated neurotoxicity syndrome (ICANS) which both can evolve in life-threatening conditions. Clinical manifestations of ICANS comprise a constellation of progressive neurological signs or symptoms which might overlap with other encephalopathies and may include aphasia, altered level of consciousness, impairment of cognitive skills, motor weakness, seizures, and cerebral oedema. With the wider implementation of CAR T-cell therapies, apart from classical ICANS new types of late neurotoxicity have emerged including movement and neurocognitive toxicities along with peripheral nerve palsies, mainly encountered with CAR-T products administered in patients with multiple myeloma (5).

Although many pre-clinical and clinical studies have investigated the pathogenesis of ICANS, the exact mechanisms driving CAR T-cell neurotoxicity remain elusive. ICANS is most frequently developed in the context of cytokine storm following CRS, however there are many cases where neurotoxicity presents as a sole entity. Previous data has shown that ICANS may be associated with increased permeability of the blood–brain barrier, endothelial activation in the central nervous system (CNS) and up-regulation of inflammatory molecules. There is also evidence of direct activity of CAR T-cells in the brain as the targeted antigens have been found in brain cells such as pericytes or basal ganglia, comprising a type of on-target off-tumor toxicity (6). This data derive mostly from the application of CAR T-cells in hematological malignancies; however, an explosion of studies are underway exploring the therapeutic potential and safety profile of CAR T-cells in solid tumors including those involving the CNS.

Therefore, there is a pressing need of better understanding the pathophysiological aspects of ICANS and late neurotoxicity in order to effectively prevent, or even to implement therapeutic strategies, as currently there are no established factors predicting neurotoxicity and no agent is completely effective at mitigating ICANS. To better tackle neurotoxicity, a multidisciplinary collaboration is needed between clinical researchers from different specialties including cell therapists, molecular engineers, hematologists, neurologists, oncologists, pediatricians and radiologists. Moreover, the contribution of scientists with a basic or translational background will substantially shed light in the field.

This Research Topic focused on the compilation of cutting edge knowledge regarding CAR T-related neurotoxicity. We welcomed articles from both basic science and clinical research investigating the pathogenesis, prediction, prevention, prognosis and treatment of ICANS in hematological malignancies, including those with primary or secondary CNS involvement, and solid tumors including CNS tumors. All submitted articles in this Research Topic underwent a rigorous peer review process and finally six articles were published.

1. The role of neurologists in the era of the growing application of novel immunotherapies and cell therapies was presented in a perspective article (Pensato, Guarino et al.). Neurologists have acquired a critical role within a multidisciplinary oncology team and training neurologists should recognize and manage novel cancer immunotherapy-related neurological syndromes, while augmenting their knowledge in neuroimmunology and neuro-oncology.

2. A case report demonstrated a novel distinctive brain feature in a patient with primary mediastinal B-cell lymphoma who developed ICANS post anti-CD19 CAR T-cell therapy (Pensato, de Philippis et al.). Reversible punctate inflammatory foci of the body and isthmus of the corpus callosum were captured in this patient in brain MRI imaging and may represent a novel radiological finding of CAR T-cell therapy-related neurotoxicity.

3. A range of potential predictive biomarkers were reviewed to better distinguish patients at increased risk for developing ICANS (Butt et al.). A framework to organize ICANS-related risk factors was presented and individual factors were broadly grouped by host, cellular therapy, and inflammatory factors.

4. Current understanding of ICANS, novel findings, and current gaps were reviewed (Genoud and Migliorini). Key mechanisms involved in the development of ICANS and strategies for ICANS management were presented.

5. Velasco et al. reviewed the currently available neurological safety data derived from clinical trials and real-world experiences in adult patients with CNS disease due to lymphoma, leukemia, or myeloma undergoing CAR T-cell therapies. Evidence was presented supporting that CNS involvement in hematologic diseases should no longer be considered an absolute contraindication to CAR T-cell therapy as while the incidence may be high, severity does not appear to be impacted significantly by pre-existing CNS status.

6. Gatto et al. reviewed the full neurotoxicity profile of CAR T-cell therapies under investigation in CNS tumors. Unlike CAR T-cell therapy in hematological malignancies, classical ICANS is not common in brain tumors: mild/moderate self-limited neurotoxicity is observed and physicians should get educated to assess individualized risk, to discern whether neurological adverse events are due to the action of the CAR-Ts or the tumor itself and provide optimal management of neurotoxicity.

We hope that this Research Topic contributes to better understanding and managing CAR T-related neurotoxicities and will also inspire researchers from a multi-disciplinary background to carry on further investigations with the aim of moving the exciting field of CAR T-cell therapy one step closer to a safer treatment modality with broader application.

## Author contributions

SB: Conceptualization, Writing—original draft, Writing—review & editing.
